# Analgesic Prescription Patterns and Pain Outcomes in Southeast Asia: Findings From the Analgesic Treatment of Cancer Pain in Southeast Asia Study

**DOI:** 10.1200/JGO.17.00055

**Published:** 2017-04-30

**Authors:** Dang Huy Quoc Thinh, Wimonrat Sriraj, Marzida Mansor, Kian Hian Tan, Cosphiadi Irawan, Johan Kurnianda, Yen Phi Nguyen, Annielyn Ong-Cornel, Yacine Hadjiat, Hanlim Moon, Francis O. Javier

**Affiliations:** **Dang Huy Quoc Thinh**, Ho Chi Minh City Oncology Hospital, Ho Chi Minh City; **Yen Phi Nguyen**, Vietnam National Cancer Hospital, Hanoi, Vietnam; **Wimonrat** **Sriraj**, Srinagarind Hospital, Khon Kaen, Thailand; **Marzida Mansor**, University of Malaya, Kuala Lumpur, Malaysia; **Kian Hian Tan**, Singapore General Hospital; **Yacine Hadjiat** and **Hanlim** **Moon**, APAC LATAM MEA, Mundipharma, Singapore; **Cosphiadi Irawan**, Cipto Mangunkusumo General Hospital, Universitas of Indonesia, Jakarta Pusat; **Johan Kurnianda**, Dr Sardjito General Hospital, Yogyakarta, Indonesia; **Annielyn Ong-Cornel**, Veterans' Memorial Medical Centre; and **Francis O. Javier**, St Luke's Medical Center, Quezon City, Philippines.

## Abstract

**Purpose:**

To identify patterns of analgesic prescription and to explore patient-reported pain intensity, sleep disturbance, and quality of life among cancer patients with pain in Southeast Asia (SEA).

**Methods:**

This cross-sectional observational study included 465 adult outpatients prescribed analgesics for cancer pain for 1 month or longer at 22 sites in Indonesia, Malaysia, Philippines, Singapore, Thailand, and Vietnam. Data on analgesic prescription and cancer characteristics were extracted from medical records. Pain intensity, sleep disturbance, and quality of life measures were recorded via questionnaires.

**Results:**

Most patients (84.4%) had stage III or IV cancer. A total of 419 patients (90.7%) were prescribed opioids; of these, 42.2% received only weak opioids, whereas 57.8% received at least one strong opioid. The mean worst pain intensity during the past 24 hours was 4.76 (standard deviation [SD], 2.47) on a scale of 0 (no pain) to 10 (worst possible pain); the mean current pain intensity was 4.10 (SD, 2.61). More than half of patients (54.8%) reported sleep disturbance caused by pain in the past 7 days. The majority of patients reported problems with pain/discomfort (82.3%), usual activities (65.8%), mobility (58.2%), and anxiety/depression (56.3%). The median daily dose prescribed in oral morphine equivalents was 30 mg for both morphine and tramadol.

**Conclusion:**

Despite unrelieved pain, sleep disturbance, and issues with quality of life, a notable proportion of patients were prescribed only weak opioids, and opioid doses prescribed were generally low. Efforts focused on encouragement of prescriptions with analgesic strength and/or doses proportional to the pain management needs of patients are vital to improve the status of cancer pain management in the region.

## INTRODUCTION

The majority of patients with cancer experience pain, and those with advanced disease stages report higher prevalence and greater intensity of pain.^1^ Pain is associated with both the disease and the treatment, and it can persist for months or years after the completion of primary adjuvant therapy.^2^

The consequences of cancer pain are well established in the literature. Pain can affect the physical and psychological well-being of patients, and it is associated with the perception of low quality of life.^3-6^ Patients with cancer have reported issues with employment, lower work performance, and sleep disturbances because of chronic pain.^5-8^ Pain can have a substantial impact on quality of life and daily activities,^3-8^ so adequate management of pain is important in both supportive and palliative care for patients with cancer.

Various international guidelines have been published to guide pain management for patients with cancer.^9-11^ Recommendations include the use of quantitative pain assessment tools and a step-up prescription of analgesics in the order of nonopioids, weak opioids, and strong opioids until adequate relief from pain is achieved.^9-11^ When applied as recommended, analgesic therapy can be effective to relieve cancer pain in most cases. However, despite broad distribution of these pain management guidelines, undertreatment of cancer pain is still widely documented.^12,13^

It is encouraging to note a general trend toward better pain management for patients with cancer in recent years. A systematic review of articles published during the last two decades reported an overall reduction in the prevalence of undertreated cancer pain from 41.5% in 2008 to 31.8% in 2014.^13^ However, the prevalence of undertreated cancer pain in Asia has remained relatively high: the weighted mean is 45.2% in Asia compared with 20.2%, 29.5%, and 32.0% in Australia, Europe, and North America, respectively.^13^ Although similar data specific to Southeast Asia (SEA) are not available, the situation is likely to be grim, because the adequacy of opioid analgesic consumption has been reported to be very low or virtually none in many SEA countries (Thailand, 1.65%; Indonesia, 0.16%; Malaysia, 3.22%; Vietnam, 0.65%; Singapore, 5.86%; Philippines, 0.45%).^14^

To better understand how cancer pain is managed in the SEA region, the analgesic treatment of cancer pain in Southeast Asia (ACE) study aimed to identify analgesic prescription patterns among cancer patients with pain in six SEA countries. This study also sought to explore patient-reported pain intensity, sleep disturbance, quality of life, levels of satisfaction with analgesic treatment, and the potential associations between these factors with the types of analgesics prescribed.

## METHODS

### Study Design and Participants

This multicenter, multinational, cross-sectional, observational study was conducted between October 2015 and December 2015 at 22 sites in six countries in SEA (Indonesia, Malaysia, Philippines, Singapore, Thailand, and Vietnam). Patients who met the eligibility criteria were recruited consecutively into the study. Inclusion criteria were as follows: age 18 years or older; pathologic diagnosis of cancer; outpatient status, with cancer pain caused by cancer itself or its treatment; and treatment with any analgesics for more than 1 month for the management of cancer pain. Exclusion criteria were as follows: had an operation for any reason within the past 3 months; an oncologic emergency; any interventional therapy (eg, nerve block, neurolytic procedures) related to cancer pain within the past 6 weeks; and current participation in any other interventional clinical trials for cancer treatment or supportive care. All patients provided written informed consent before study enrolment.

All study data were collected in a single visit via patient medical records and questionnaires. The primary variables of interest were analgesic prescription patterns, quality of life, pain intensity (current pain intensity and worst pain intensity during the past 24 hours), and satisfaction with pain control. Secondary end points included sleep disturbance because of pain in the past 7 days, Eastern Cooperative Oncology Group (ECOG) grade, and cancer-related information. The study protocol, case report forms, and documents used for informed consent were reviewed and approved by the local ethics committee at each study site. All study procedures were conducted in accordance with the Declaration of Helsinki and in compliance with local regulatory requirements.

### Study Assessments

Patient medical records were reviewed to extract data about demographics, cancer characteristics, treatment histories, and current analgesic prescriptions. Patient questionnaires were administered to assess pain intensity (scored on a numeric rating scale from 0 [no pain] to 10 [worst pain imaginable]^15,16^), sleep disturbance caused by cancer pain within the last 7 days, quality of life (evaluated with the EuroQol Group 5-dimension self-report questionnaire 3-level [EQ-5D-3L] system), and patient satisfaction with pain control status (scored on a five-point scale: very satisfied, satisfied, acceptable, dissatisfied, very dissatisfied). Investigators evaluated patient performance statuses with the ECOG grading scale (scored on an ordinal scale of 0, 1, 2, 3, and 4 [lower grades indicate higher level of functioning]) and recorded sites of pain for each patient.

### Statistical Analyses

This was an exploratory study, so statistical sample size estimation was not performed. The study included 465 patients from six countries in SEA (n = 81 from Indonesia, n = 100 from Malaysia, n = 105 from the Philippines, n = 8 from Singapore, n = 100 from Thailand, and n = 71 from Vietnam); 462 patients fulfilled the eligibility criteria and were included in the analyses. Patient demographics, cancer characteristics, treatment histories, ECOG grades, sites of pain, pain intensities, EQ-5D-3L responses, and satisfaction with pain control were summarized with descriptive statistics. Continuous variables were expressed as the mean (standard deviation [SD]), and categoric variables were expressed as the number (percentage).

Patients subsequently were grouped into three groups according to the type of analgesics prescribed: nonopioid only, opioid only, or nonopioid and opioid. Differences between groups in terms of cancer stage, treatment, pain intensity, sleep disturbance, EQ-5D-3L, and satisfaction with pain control were analyzed by using one-way analysis of variance for continuous variables or χ^2^ test and Fisher’s exact test for categoric variables. All variables with univariable *P* values of < .10 were included in subsequent multivariable regression models. The associations between variables and analgesic type prescribed were tested with multiple logistic regression or multiple linear regression that controlled for potential confounders. *P* values < .05 were considered statistically significant. All statistical analyses were performed with R, version 3.1.3 (R development core team, Vienna, Austria).

## RESULTS

### Demographics and Baseline Characteristics

A total of 465 patients were enrolled in the study. Of these, three patients did not fulfill the eligibility criteria and were excluded from the analysis; 462 patients remained in the analysis population.

Demographics and baseline characteristics of the analysis population are listed in Table 1. The mean age of patients was 55.14 years (SD, 13.39 years), and most (54.6%) were between 50 and 69 years old (Table 1). Both sexes were well represented in the analysis population: 46.3% were men, and 53.7% were women. The majority of patients included in the analysis had stage III (17.1%) or stage IV (67.3%) cancer; 65.6% had metastatic disease; and 93.1% had received surgery, radiotherapy, or chemotherapy (Table 1). The most common cancer types were breast cancer (23.6%) and lung cancer (11.5%).

**Table 1:**
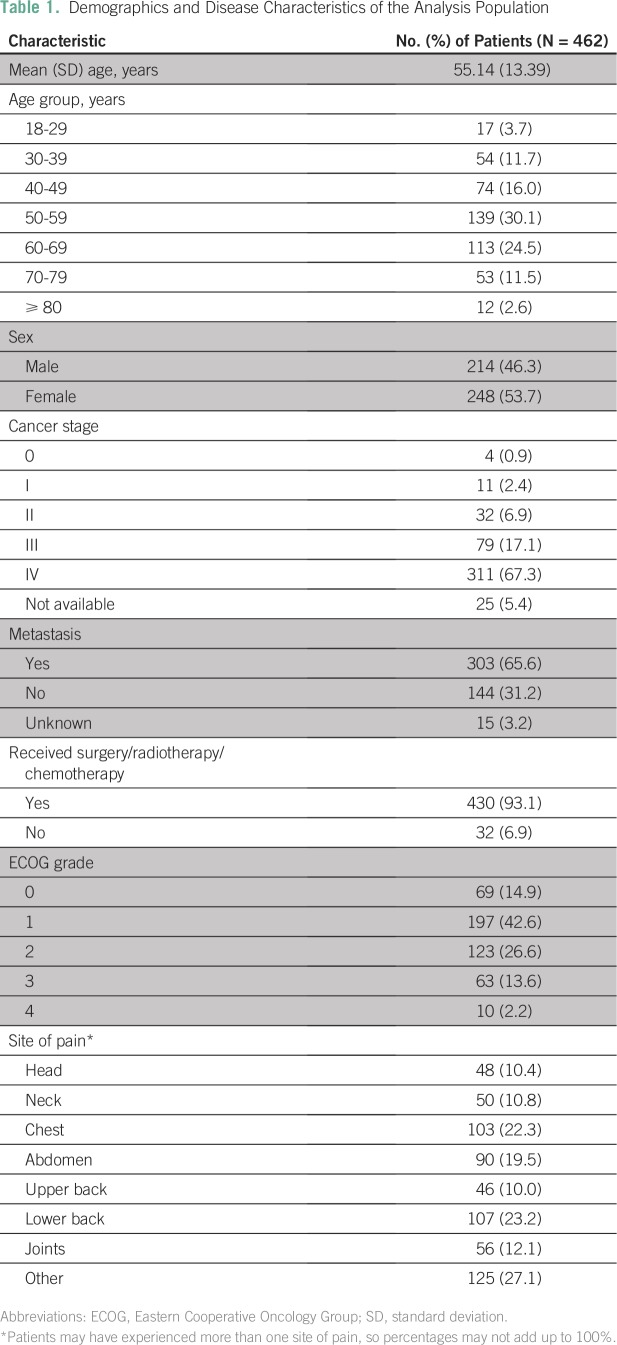
Demographics and Disease Characteristics of the Analysis Population

Overall, the majority of patients had an ECOG grade of 2 or less (ECOG grades 0, 1, and 2: 14.9%, 42.6%, and 26.6%, respectively). The most common sites of pain were lower back (23.2%), chest (22.3%), and abdomen (19.5%). More than a quarter of patients (27.1%) had pain at other sites (Table 1).

### Analgesic Prescription Patterns

**Fig 1:**
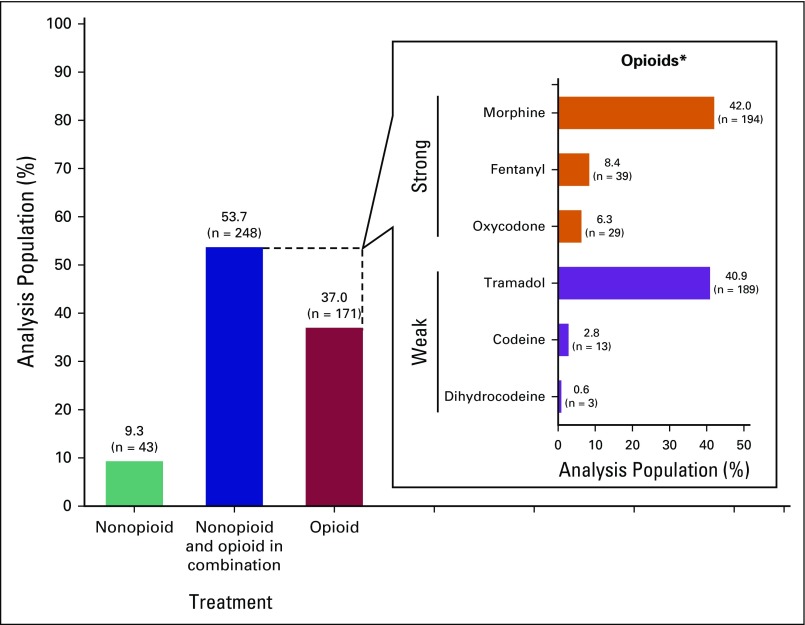
Analgesic prescription patterns in the analysis population (N = 462). (*)Patients may be prescribed one or more opioids.

More than half of the analysis population (53.7%; n = 248) received a combination of nonopioid and opioid analgesics for cancer pain (Fig 1). A smaller proportion of patients were prescribed only opioid analgesics (37.0%; n = 171) or only nonopioid analgesics (9.3%; n = 43).

Of the 419 patients (90.7%) who were prescribed opioid analgesics (either alone or in combination with nonopioid analgesics), 42.2% received only weak opioids, whereas 57.8% received at least one strong opioid. Morphine was the most common strong opioid prescribed (42.0%; n = 194), and tramadol was the most common weak opioid prescribed (40.9%; n = 189; Fig 1).

Weighted estimates of the overall prescription rates in the participating countries in SEA were 11.33% (95% CI, 7.58% to 15.31%) for only nonopioid analgesics prescription, 37.27% (95% CI, 31.83% to 42.92%) for only opioid analgesics prescription, and 51.40% (95% CI, 46.13% to 56.83%) for prescription of both nonopioid and opioid analgesics. See the Data Supplement for a summary of the guidelines used for cancer pain management in the participating centers within the six countries.

### Pain Intensity, Sleep Disturbance, Quality of Life, and Satisfaction With Pain Control

On a numeric rating scale from 0 (no pain) to 10 (worst pain imaginable), the mean current pain intensity was 4.10 (SD, 2.61), and the mean worst pain intensity during the past 24 hours was 4.76 (SD, 2.47; Table 2). More than half of patients (54.8%) reported sleep disturbance because of pain in the past 7 days.

**Table 2:**
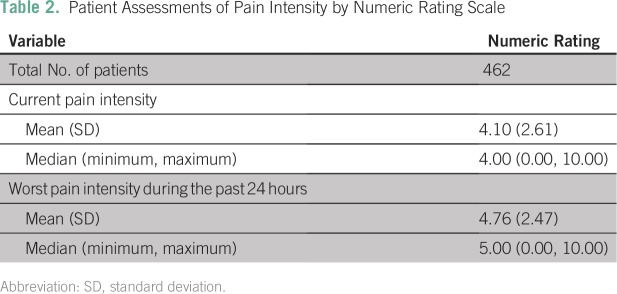
Patient Assessments of Pain Intensity by Numeric Rating Scale

The majority of patients reported problems with pain/discomfort (82.3%), usual activities (65.8%), mobility (58.2%), and anxiety/depression (56.3%; Table 3). However, most had no problems with self-care (60.8%). The mean EQ-5D-3L summary index was 0.45 (SD, 0.30), and the median value was 0.51 (range, −0.45 to 0.80; Table 3). The mean EuroQol visual analog score (EQ-VAS) value was 61.55 (SD, 20.18), which indicated a moderate state of health (Table 3).

**Table 3:**
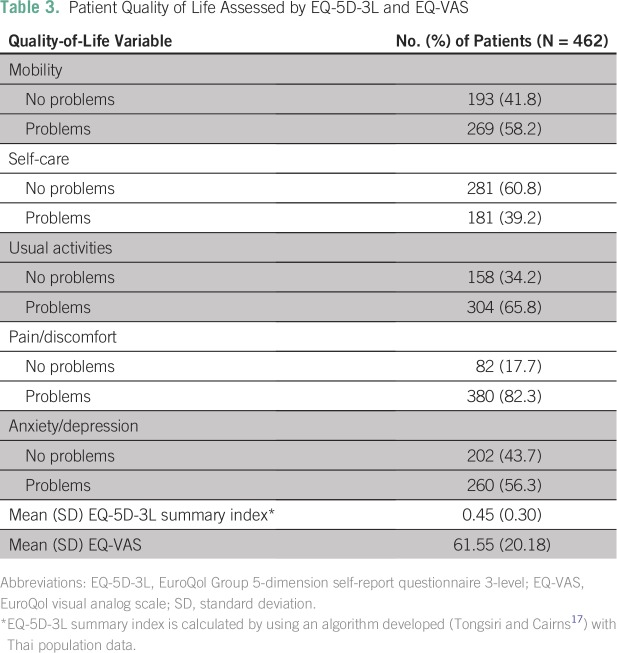
Patient Quality of Life Assessed by EQ-5D-3L and EQ-VAS

More than half of patients indicated satisfaction with their pain control status. A total of 192 (41.6%) were satisfied, and 86 (18.6%) were very satisfied.

### Association of Cancer Stage, Treatment, Pain Intensity, Sleep Disturbance, EQ-5D-3L, and Satisfaction With Pain Control by Analgesic Type

The type of analgesic prescribed (nonopioid, opioid, nonopioid and opioid) was significantly associated with cancer stage (*P* = .019), metastasis (*P* = .016), surgery/radiotherapy/chemotherapy treatment (*P* = .049), concurrent complementary pain therapy (*P* < .001), and EQ-5D dimension of pain/discomfort (*P* = .046) and of anxiety/depression (*P* = .039; Table 4). However, the type of analgesic prescribed was not significantly associated with pain intensity, sleep disturbance, EQ-VAS, or patient satisfaction with pain control (Table 4).

**Table 4:**
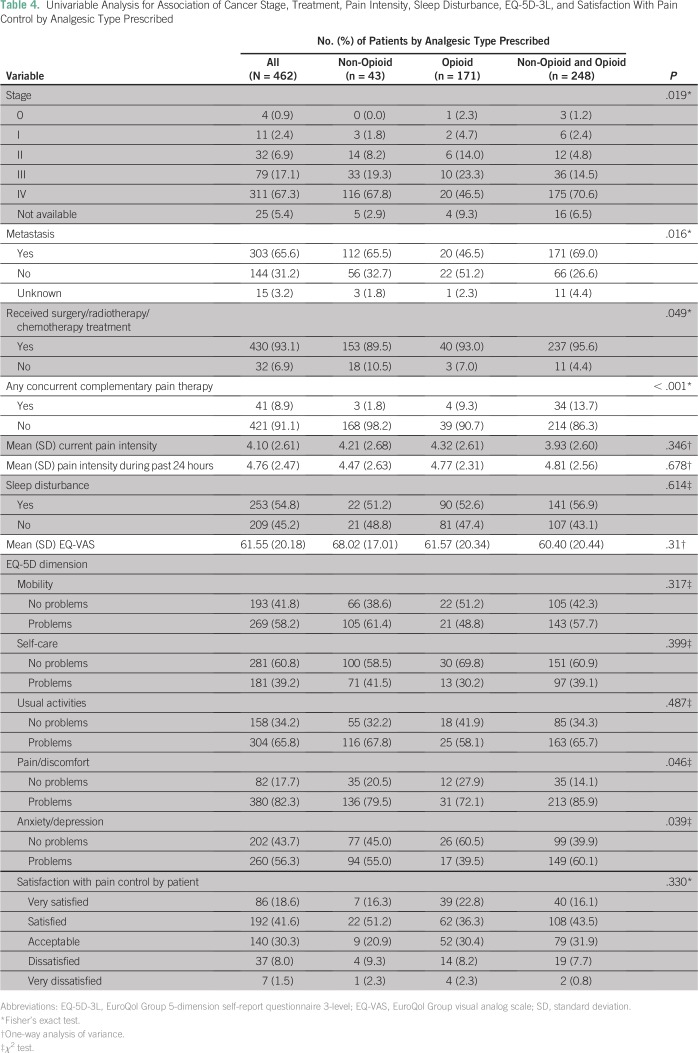
Univariable Analysis for Association of Cancer Stage, Treatment, Pain Intensity, Sleep Disturbance, EQ-5D-3L, and Satisfaction With Pain Control by Analgesic Type Prescribed

In multivariable analyses that adjusted for potential confounders, only the EQ-5D-3L dimension of anxiety/depression was associated with type of analgesic prescribed. The odds of reported problems in anxiety/depression in the EQ-5D-3L questionnaire were 2.14-fold (95% CI, 1.10-fold to 4.28-fold) higher for patients prescribed a combination of nonopioid and opioid compared with patients prescribed only nonopioids (*P* = .028).

### Doses of Analgesics Prescribed

The median daily dose of morphine prescribed was 30.00 mg (range, 2.00 to 300.00 mg). The median daily dose of tramadol prescribed was 150.00 mg (range, 30.00 to 420.00 mg), which was equivalent to 30.00 mg (range, 6.00 to 84.00 mg) of oral morphine (Data Supplement).

## DISCUSSION

Current data available in the literature on cancer pain management and practices in SEA are mostly country specific.^18-20^ To our knowledge, the ACE study is the first multinational prospective study conducted in SEA to identify analgesic prescription patterns among patients with cancer and the relationship of these patterns with pain outcomes. In this study, the cancer characteristics, treatment histories, and prescribed analgesic types and doses were obtained directly from patient medical records. In addition, established tools were used to assess pain intensity (numeric rating scale) and quality of life (EQ-5D-3L). More than 80% of patients in this study had stage III or IV cancer. Overall, nine in 10 patients were prescribed opioids for cancer pain; however, a significant proportion of these patients were prescribed only weak opioids. Although the majority of patients indicated satisfaction with respect to pain control, most still reported unrelieved pain, problems with pain/discomfort, quality-of-life issues, and sleep disturbance because of cancer pain. Surprisingly, receipt of opioids was not associated with significantly different pain intensity and odds of quality-of-life problems.

Despite active analgesic treatment, many patients with cancer in the ACE study still experienced pain; the mean reported pain level was moderate intensity (current pain intensity, 4.10 [SD, 2.61]; pain intensity in the past 24 hours, 4.76 [SD, 2.47]). One possible reason for this observed undermanagement of pain may be the lack of use of objective pain scales (eg, the 10-point numeric rating scale used in the ACE study) to assess and monitor pain on a routine basis in Asia.^6,21-24^ Almost half of patients in the recent ACHEON (Current Practices of Cancer and Chronic Noncancer Pain Management) survey reported that pain was assessed subjectively without the use of a quantitative pain scale.^6^ The lack of objective pain assessment practices may stem from inadequate physician training in pain assessment methods^25^ and/or limited ability of patients to understand or use pain scales effectively. Because pain is a subjective experience, it is important that analgesic therapy for cancer pain management be individualized and guided by patient-reported intensity of pain.^26^ Comprehensive pain assessment techniques, which take psychological distress and quality-of-life measures into account, may help capture the multidimensional nature of cancer pain^27^; however, these are usually more complex to administer. More initiatives are needed to standardize pain assessment approaches across SEA and to enhance both patient and physician competency in effective reports and assessments of pain.

Although greater than 90% of patients in the ACE study were prescribed opioids for cancer pain management, the majority still reported sleep disturbance because of pain and problems with quality of life (eg, pain/discomfort, usual activities, mobility, and anxiety/depression). Patient-reported quality of life may be confounded by cancer-related anxiety or depression,^28^especially because the majority of patients were diagnosed with late-stage cancer and had metastatic disease. Moreover, greater than 90% of patients had received treatment of their cancer (eg, surgery, radiotherapy, or chemotherapy), and adverse effects from these treatments may affect perceived quality of life.^28^

Notably, even when multivariable analyses were adjusted for potential confounders, opioid prescription was not associated with lower pain scores, lesser sleep disturbance because of pain, better quality of life, or more satisfaction with pain control. When the between-group differences were significant, the group that received opioids had higher odds of problems with quality of life. Although the specific reasons for patient-reported problems with quality of life were not explored in this study, we speculate that undermanaged opioid-related adverse effects may be a plausible source of these quality-of-life issues. Opioid use is known to be accompanied by adverse effects and complications, such as constipation, nausea, and sedation, which can significantly impair quality of life and affect treatment compliance.^29,30^ Often, adverse effects can limit the doses of opioids that the patient is able to tolerate. Symptomatic management of these adverse effects is commonly applied in clinical practice; however, many of the agents used have their own adverse effect profiles as well.^31^ Other recommended strategies include opioid dose reduction, opioid rotation, and changes to the route of systemic administration.^31,32^ A careful review of predisposing patient factors, comorbidities, and performance status may aid clinicians in selection of a strategy to manage opioid-induced adverse effects and to maximize the effectiveness of opioids but reduce the impact of the adverse effects on patient quality of life.

The majority of cancers in low-income and middle-income Asian countries are diagnosed in advanced cancer stages.^33,34^ In this study, more than 80% of the patients had either stage III or IV cancer. Patients in advanced cancer stages tend to suffer from greater intensity of pain and, in most instances, require strong opioids for adequate relief of cancer pain.^1,9-11^ However, many of the participating SEA countries have considerable regulatory restrictions on the use of strong opioids, which include (1) limited duration of opioid prescription (Indonesia, a few days; Vietnam, 10 days; Philippines and Thailand, 1 month), (2) burdensome procedures to report opioid prescriptions (Indonesia), (3) complex procedures to obtain a license to prescribe opioids (Indonesia, Vietnam, Philippines), and (4) excessively restrictive policies that govern the use of opioids (Vietnam and Philippines).^24^ These regulatory barriers may lead to physician reluctance to prescribe strong opioids or to prescribe an adequate dose proportional to pain intensity of patients. In addition, affordability issues in SEA (eg, limited health insurance coverage of opioids) may affect both prescription and consumption patterns of opioids and may limit accessibility to strength and/or doses of opioids that meet pain management needs for patients.^35-38^ Indeed, although most patients in the ACE study had late-stage cancer and reported unrelieved pain and problems with pain/discomfort, a notable proportion received only weak opioids, and opioid doses prescribed were generally low (median daily dose of morphine tramadol, 30.00 mg oral morphine equivalent). Although regulatory restrictions were intended to prevent opioid abuse, their profound effects on prescribing practices, pain management, and patient quality of life should not be overlooked and must be addressed.

As with most patient-reported outcomes, pain score, quality of life, and satisfaction with pain control are subjective and may be affected by sociocultural differences between countries. Furthermore, confounding factors related to cancer and/or its treatment (eg, psychological distress, adverse events) on patient-reported outcomes were not captured in this cross-sectional study. Importantly, study sites included in the ACE study were large hospitals situated in the major cities of participating SEA countries; thus, interpretation of the study findings may be limited to more developed regions of SEA. Undermanagement of cancer pain is expected to be even more severe in less-developed regions of SEA, where accessibility to pain management specialists and analgesics is limited.

The majority of cancer pain in patients in this study from the participating SEA countries were prescribed opioids (often in combination with nonopioids), although a notable proportion of patients were prescribed only weak opioids. Despite active analgesic treatment, many patients still experienced unrelieved pain, problems with pain/discomfort, quality-of-life issues, and sleep disturbance because of pain. Notably, management with opioids was not associated with better patient outcomes (ie, lower pain scores or better quality of life). The ACE study results suggest suboptimal cancer pain management in SEA. To address this issue, pain should first be assessed systematically through application of standardized pain assessment techniques. Next, beyond the prescription of analgesics, particular attention should be paid to the appropriate selection of analgesics (eg, strong *v* weak opioids) and to the prescription of analgesic doses that provide adequate pain control. Finally, effective assessment and proactive management of adverse effects are needed to support analgesic compliance and to improve patient quality of life.
